# Structured Supportive Dentistry Intervention Manual for the Oral Complications in Patients With Cancer Receiving Chemotherapy: Development and Content Validation

**DOI:** 10.1155/ijod/1554891

**Published:** 2026-08-07

**Authors:** Nakshatra Shetty, Arun Ghoshal, Adarsh Kudva, Ananth Pai, Ravindranathan Vineetha, Sudeendra Prabhu, Anuja Damani, Krithika S. Rao, Abhinav Mishra, S. Gayatri, Kunal Das, Naveen Salins

**Affiliations:** ^1^ Department of Palliative Medicine and Supportive Care, Kasturba Medical College, Manipal Academy of Higher Education, Manipal, India, manipal.edu; ^2^ Department of Oral and Maxillofacial Surgery, Manipal College of Dental Sciences, Manipal Academy of Higher Education, Manipal, India, manipal.edu; ^3^ Department of Medical Oncology, Kasturba Medical College, Manipal Academy of Higher Education, Manipal, India, manipal.edu; ^4^ Department of Oral Medicine and Radiology, Manipal College of Dental Sciences, Manipal Academy of Higher Education, Manipal, India, manipal.edu; ^5^ Department of Oral Pathology, Yenepoya Dental College, Yenepoya (Deemed to be University), Mangalore, Karnataka, India, yenepoya.edu.in

**Keywords:** chemotherapy, content validation, oral complications, oral mucositis, supportive dentistry

## Abstract

**Introduction:**

Chemotherapy‐related oral complications (CROC), such as oral mucositis (OM), xerostomia and candidiasis, affect 40%–80% of patients with cancer in India, compromising quality of life (QOL) and treatment outcomes. Standardised, evidence‐based, supportive dentistry intervention manuals (SDIM) tailored to local contexts remain limited. This study aims to develop and evaluate the content validity, clinical relevance, clarity and usability of a SDIM for managing CROC in patients with cancer receiving chemotherapy (CT), as assessed by expert dental clinicians.

**Methods:**

The manual was developed through a comprehensive review of literature, clinical guidelines and oncology oral care resources, followed by contextual adaptation. It was structured into four domains: (1) basic oral care, (2) soft tissue‐related complications, (3) oral infections and (4) hard tissue complications. Content validation was conducted using a content validity index (CVI)–based approach informed by Lawshe’s framework, complemented by qualitative expert feedback to refine and validate the structured SDIM. Ten purposively selected senior dental experts (at least 5 years of experience) from the Department of Oral Medicine and Radiology evaluated the manual using a 4‐point Likert scale for relevance, clarity, simplicity and necessity.

**Result:**

Experts found the manual clinically useful and relevant for managing common CROC, particularly xerostomia, OM and candidiasis. Feedback highlighted the need to simplify for multidisciplinary use, improve structuring for quick reference, and include context‐specific, feasible interventions. Suggestions also included reducing dense text and standardising tables and flowcharts for better usability.

**Conclusion:**

The manual was considered relevant, practical and appropriate. Clinical piloting remains a necessary next step before formal implementation recommendations can be made.

## 1. Introduction

Chemotherapy (CT) plays an important role in the management of cancer worldwide (50%–70%), with over one million patients in India annually receiving it as either a first‐line or adjuvant therapy for solid and haematological malignancies [1]. Although CT regimens achieve remission rates of 30%–60%, they often cause dose‐limiting chemotherapy‐related oral complications (CROC) [2].

CROC are experienced by 40%–80% of patients undergoing CT, and commonly includes oral mucositis (OM) (20%–40%) [3], xerostomia, dysgeusia, opportunistic infections and medication‐induced osteonecrosis of the jaw (MRONJ). These complications result from mucosal cytotoxicity, salivary gland dysfunction and immunosuppression, peaking at 7–14 days after CT initiation [4]. CROC significantly impair quality of life (QOL), leading to nutritional issues and deterioration of oral health‐related QOL [5]. Importantly, severe oral complications may require CT dose reduction, treatment delay or discontinuation, potentially affecting therapeutic outcomes and increasing healthcare costs [6].

Although several international guidelines provide evidence‐based recommendations for the prevention and management of CROC [6–8], translating these recommendations into routine clinical practice remains challenging, particularly in low‐resource oncology settings. In India, factors such as high patient loads, limited integration of dental professionals within oncology teams, fragmented referral pathways and cost‐related barriers often impede the delivery of systematic supportive oral care [7, 8]. To our knowledge, no structured, implementation‐focused supportive dentistry manuals have been developed in India to assist oncologists in making quick chairside assessments and management of CROC. A structured, simplified and contextually adaptable manual is necessary to translate evidence‐based recommendations into practical, actionable steps suitable for routine oncology practice [8]. The supportive dentistry intervention manual (SDIM) was developed to address this crucial gap by providing a generalised yet adaptable clinical approach that enables quick assessment, structured management, and clear referral pathways across diverse clinical settings, including those with limited resources [9].

To address this gap, a structured SDIM was developed and underwent a methodological content validation process combining quantitative content validity assessment and qualitative expert review. Ten experts rated all 13 components on a four‐point Likert scale, yielding an item‐level content validity index (I‐CVI) ≥0.80 and a scale‐level content validity index (S‐CVI/Ave) of 0.93 after refinement. This systematic validation confirms the manual’s clarity, relevance, expert‐perceived feasibility and adaptability to clinical practice in Indian oncology settings.

## 2. Methods

### 2.1. Study Design

This methodological study evaluated the content validity of the SDIM using a quantitative expert‐validation approach complemented by qualitative feedback, such as structured clinical protocol evaluations reported in healthcare settings [1]. Content validation was conducted using a methodological approach informed by Lawshe’s content validity framework and adapted to employ the I‐CVI and S‐CVI/Ave, as operationalised by Lynn [10] and Polit and Beck [11], to ensure the manual’s clinical relevance, clarity and expert‐perceived feasibility [2].

**Figure 1:**
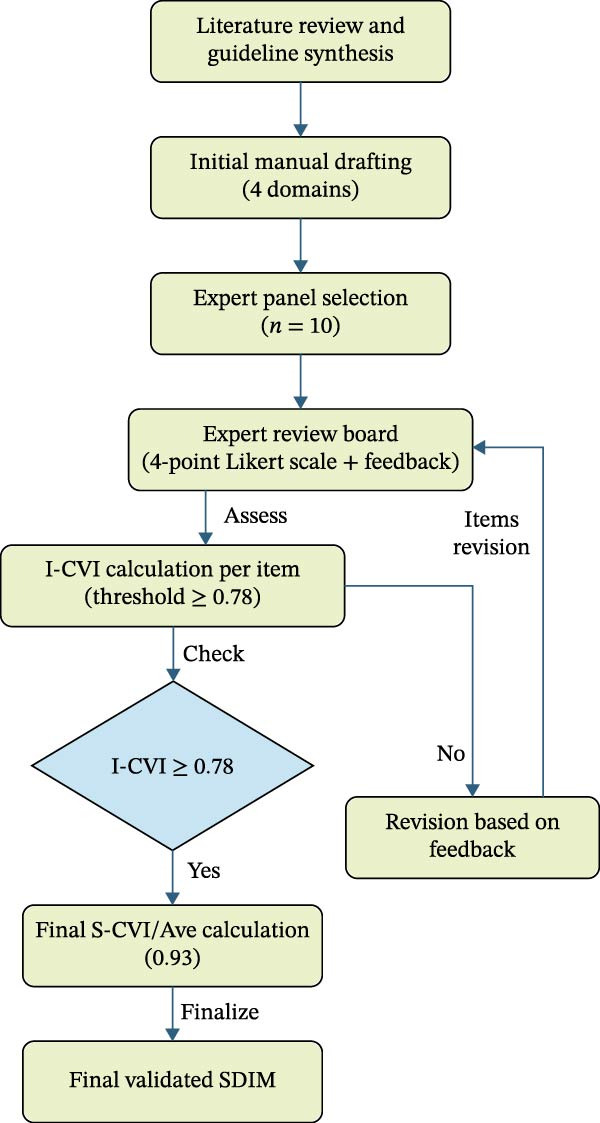
The flowchart illustrates the SDIM development and content validation process.

The stepwise development and validation process is summarised in Figure 1.

### 2.2. Development of the Manual

The SDIM was developed by synthesising evidence from published scientific literature, established clinical practice guidelines and oncology‐specific oral care resources related to CROC. Literature was identified using databases such as PubMed, Scopus, and Google Scholar using keywords including “chemotherapy”, “oral complications”, “oral mucositis”, “xerostomia”, “oral candidiasis”, “supportive oral care”, “dental care in cancer patients” and “clinical guidelines”. In addition, established guidelines such as those from the Multinational Association of Supportive Care in Cancer and the International Society of Oral Oncology (MASCC/ISOO) and institutional oral care manuals were referred [4–8]. Evidence from published guidelines and peer‐reviewed literature was critically appraised and synthesised to construct a clinically applicable manual suited to Indian oncology practice.

Based on this, the manual was organised into four key domains: (1) basic oral care protocols, (2) soft tissue complications, (3) oral infections and (4) hard tissue‐related complications. Each section included structured assessment criteria, identification of common clinical presentations, and corresponding management strategies. The content was designed to be clear, concise and clinically applicable, facilitating its use by multidisciplinary oncology teams. Adaptations were also made to ensure feasibility within the Indian oncology setting, considering resource availability and practice variations.

**Figure 2:**
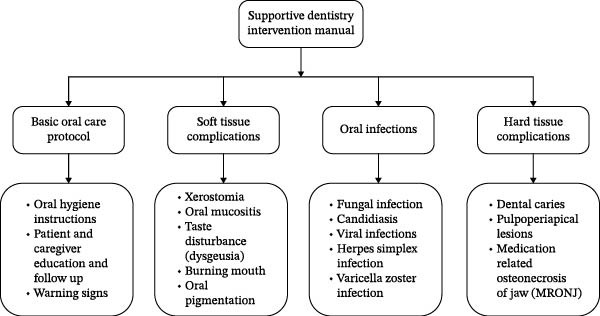
Structural overview (Four domain framework).

A schematic overview of the manual’s four‐domain structure is presented in Figure 2. Representative sections of the SDIM, including the content, sample management table and sample flowchart for the basic oral care protocol, are provided in Tables S1 and S2 and Figure S1, respectively.

### 2.3. Study Questionnaire

A structured validation questionnaire was developed to evaluate each component of the manual across four domains: relevance, clarity, simplicity and necessity [9, 12]. Items were rated using a four‐point Likert scale (1 = not relevant, 2 = needs major revision, 3 = relevant but needs minor revision, 4 = highly relevant). Experts were also invited to provide qualitative feedback through open‐ended comments to inform modifications or improvements to the manual.

### 2.4. Participants’ Recruitment, Sampling and Sample

A purposive homogenous sampling strategy was employed to recruit subject experts for content validation [12]. Eligible participants were dental specialists in oral medicine with at least 5 years of clinical and/or academic experience in managing oral complications associated with oral cancer and cancer therapies. Ten experts meeting the eligibility criteria were invited to participate. Experts were recruited from four academic institutions, including Manipal Academy of Higher Education, Manipal and Mangalore; A.J. Institute of Dental Sciences, Mangalore; and Keele University, Sharjah. The expert panel size (*n* = 10) aligns with methodological recommendations by Lynn [10], Polit and Beck [11], and Romero Jeldres et al. [2], who suggested that 5–10 subject matter experts are sufficient to obtain reliable estimates of content validity. An I‐CVI threshold of ≥0.78 is regarded as acceptable to ensure agreement beyond chance.

### 2.5. Data Collection

The SDIM was circulated electronically to experts for independent review. Participants provided suggestions and comments directly within the document. Following the review, experts completed the validation questionnaire and provided additional qualitative feedback on the manual’s clarity, relevance and feasibility.

### 2.6. Data Analysis

Content validity was evaluated using Lawshe’s content validity framework, and I‐CVI and S‐CVI/Ave were calculated according to the approach described by Lynn [10] and Polit and Beck [11]. This approach is widely adopted in instrument and protocol validation studies because it enables systematic evaluation of item relevance using expert ratings obtained on a four‐point Likert scale [13].

For a panel of 10 experts, an I‐CVI ≥ 0.78 was considered indicative of acceptable content validity. The S‐CVI was calculated as the average of all I‐CVI values. Components that did not meet the predefined criterion were revised based on the expert’s qualitative feedback before being retained in the final version of the manual.

To account for the agreement occurring by chance, the modified kappa (*k*) statistics were calculated for each item, using the formula:

k=I-CVI−Pc/1−Pc∗,

where *P*_c_ represents the probability of chance agreement among experts. *P*_c_ is calculated by using the formula:

Pc=N!/A!N−A!×0.5N,

where *N* = number of experts = 10, *A* = number of experts who rated ≥3 (that is, the numerator of I‐CVI), 0.5 = probability of rating relevant versus not relevant by chance [14]. All items yielded *k* values more than 0.74, indicating excellent agreement beyond chance.

## 3. Result

A total of 10 experts completed the content validation process. All 13 components of the SDIM were evaluated across four domains for clarity, simplicity, and necessity. Overall, most items were rated highly relevant, clear, practical and essential for supportive oral care in patients undergoing CT. Expert feedback primarily led to minor structural refinements and targeted content modifications to enhance clinical usability, with no major deletions to the foundational framework.

### 3.1. Basic Oral Care Protocol

The oral hygiene instructions and the patients’ and caregivers’ education components were unanimously rated as highly relevant. Key refinements included repositioning this section to the start of the manual, revising toothbrush replacement frequency from monthly to every 3 months, adding instructions for waxed dental floss, adding tongue cleaning instructions, incorporating alcohol‐free 0.2% chlorhexidine mouthwash for gingivitis management, and introducing a daily oral care checklist with a 1‐month follow‐up. No items were removed; only minor structural refinements were made to improve clarity and patient‐centeredness. Sample management for oral hygiene is provided in Table S2, and the flowchart illustrating the basic oral care protocol is provided in Figure S1.

### 3.2. Soft Tissue Complications

OM, xerostomia and burning mouth achieved perfect expert agreement (I‐CVI = 1.00). The mucositis risk assessment table was categorised from low to high severity, and the World Health Organisation (WHO) analgesic ladder was incorporated for pain management guidance. For xerostomia, saliva collection timing was standardised to minimise circadian variation bias. Dysgeusia content was refined by removing overlap with the nutritional assessment and by expanding the dietary recommendations. Capsaicin was proposed for burning mouth but was excluded due to insufficient oncology‐specific evidence. Photographic documentation was added to monitor oral pigmentation.

### 3.3. Oral Infections

All components of oral infections were rated as relevant and appropriate. Clinical grading criteria for candidiasis were more clearly defined, and herpes simplex and varicella zoster content were restructured into a tabular format for quick clinical reference.

### 3.4. Hard Tissue Complications

The Caries Management by Risk Assessment (CAMBRA) approach and the periapical granuloma subsection were removed because more than 50% of experts considered them less relevant in the oncology setting. Explicit referral recommendations to dental specialists were retained and strengthened. The MRONJ section was deemed highly relevant; the risk factor list was removed to maintain a management‐focused structure.

Overall, expert feedback led to targeted structural improvements, better standardisation and clearer clinical guidance. No significant deletions were needed, and all essential components remained in the finalised manual. A summary of modifications following expert review is presented in Table 1.

**Table 1:** Summary of modifications following expert review.

Section	Original item	Modification made	Justification
Basic oral care	Toothbrush replacement monthly	Revised every 3 months	Standard preventive dentistry guidance
Oral mucositis	No analgesic ladder	WHO analgesic ladder added	Strengthened pain management guidance
Xerostomia	No saliva timing mentioned	Timing standardised	Reduce circadian variation bias
Dysgeusia	BMI under dysgeusia	Shifted to the assessment section	Avoid redundancy
Dental caries	Included CAMBRA	Removed	Maintain feasibility in the oncology setting
Pulpoperiapical	Periapical granuloma listed	Removed	Limited clinical distinctiveness
MRONJ	Detailed risk factors are listed	Removed	Maintain management‐focused structure
Infections	Narrative description	Tabulated format	Improve quick reference usability

The I‐CVI for each component of the manual demonstrated strong agreement among experts, with values ranging from 0.80 to 1.00. Components such as patient and caregiver education, xerostomia, OM, burning mouth, candidiasis, and MRONJ achieved perfect agreement (I‐CVI = 1.00). Oral hygiene instructions, dysgeusia, oral pigmentation, varicella zoster infection and pulpoperiapical lesions showed high content validity with I‐CVI values of 0.90. Herpes simplex infection and dental caries demonstrated slightly lower, yet acceptable agreement, with I‐CVI values of 0.80. These findings indicate that all components met the acceptable threshold for content validity. I‐CVI values represent the proportion of experts who rate each item as 3 (relevant but needs minor revision) or 4 (highly relevant) on the four‐point Likert scale. The detailed I‐CVI values and modified *K* for each domain are presented in Table 2, and the interpretation of the Kappa value is presented in Table 3.

**Table 2:** I‐CVI and modified Kappa (*k*  ^∗^) of manual components (*n* = 10 experts).

Domain	Content area	No. of experts rating ≥3 (relevant/highly relevant)	I‐CVI	Modified kappa (k)
Basic oral care protocol	Oral hygiene instructions	9	0.90	0.90
Basic oral care protocol	Patient and caregiver education	10	1.00	1.00
Soft tissue complications	Xerostomia	10	1.00	1.00
Soft tissue complications	OM	10	1.00	1.00
Soft tissue complications	Dysgeusia	9	0.90	0.90
Soft tissue complications	Burning mouth	10	1.00	1.00
Soft tissue complications	Oral pigmentation	9	0.90	0.90
Oral infections	Candidiasis	10	1.00	1.00
Oral infections	Herpes simplex infection	8	0.80	0.79
Oral infections	Varicella zoster infection	9	0.90	0.90
Hard tissue complications	Dental caries	8	0.80	0.79
Hard tissue complications	Pulpoperiapical lesions	9	0.90	0.90
Hard tissue complications	MRONJ	10	1.00	1.00

*Note:* I‐CVI = proportion of experts rating the item 3 or 4 on the four‐point Likert scale (1 = not relevant, 2 = needs major revision, 3 = relevant but needs minor revision, 4 = highly relevant). Threshold for acceptability: I‐CVI ≥ 0.78 for a 10‐expert panel. Modified *k* calculated using formula: *k* = (I‐CVI−*P*_c_)/(1−*P*_c_) ^∗^, where *P*_c_ = probability of chance agreement. *k* ≥ 0.74 = excellent agreement.

**Table 3:** Interpretation of kappa value [3, 14].

*k*‐value	Interpretation
>0.74	Excellent
0.60–0.74	Good
0.40–0.59	Fair
<0.40	Poor

The overall S‐CVI/Ave of the manual was 0.93, indicating strong content validity. A total of 13 items were evaluated, and all met the recommended validity criteria. These results confirm strong overall agreement among experts regarding the relevance and adequacy of the manual content. The summary of S‐CVI/Ave is presented in Table 4.

**Table 4:** S‐CVI of the manual.

Measure	Value
Total I‐CVI	12.1
Number of items evaluated	13
Range of I‐CVI	0.80–1.00
S‐CVI/Ave	0.93 (Total I‐CVI/number of items evaluated)
Interpretation	Excellent content validity

## 4. Discussion

The study developed and content‐validated a structured SDIM for managing CTOC. This manual aims to provide practical, evidence‐based oral care for oncology settings [15]. Evaluation by the expert panel demonstrated strong agreement on the manual’s relevance, clarity, simplicity and necessity, supporting its suitability as a clinical resource. Although all items met the acceptable I‐CVI threshold, agreement was comparatively lower for herpes simplex infection (0.80) and dental caries (0.80). Experts raised feasibility concerns about the CAMBRA approach and periapical granuloma, given their reduced relevance in the manual. Overall, structural refinements were introduced to improve clarity and the feasibility of implementation. The highest agreement was observed for OM, xerostomia and candidiasis (I‐CVI = 1.00), reflecting the greatest clinical burden in patients receiving CT.

The validation process yielded primarily structural refinements rather than conceptual revisions, suggesting that the manual’s foundational framework was robust. Items that required modification were revised to enhance feasibility in the Indian oncology setting. For example, the reordering of sections, the addition of the WHO analgesic step ladder, and the removal of periapical granuloma and CAMBRA. The CAMBRA approach was removed during expert refinement because it was deemed too time‐consuming and documentation‐intensive for chairside use by non‐dental clinicians in a high‐volume setting. In its place, the manual retains a simplified caries risk indicator that oncology clinicians can apply rapidly, with mandatory dental referral for at‐risk patients for more detailed risk stratification, preserving feasibility without eliminating structured caries risk assessment. These refinements ensured that the manual remained practical, clinically coherent and implementable without increasing clinical workload [13].

The SDIM was developed using recommendations from established resources, including the MASCC/ISOO guidelines and the PDQ guidelines, which represent the gold standard of evidence‐based recommendations for managing treatment‐related CROC; however, these documents are structured as consensus statements intended to guide specialist clinical decision‐making and are not designed as streamlined, workflow‐integrated tools for rapid chairside use [4–7, 16–18]. Similarly, the BC cancer oral care manual offers comprehensive protocols within an established dento‐oncology framework but is tailored for healthcare systems with integrated oral oncology services, dedicated dental oncology teams, and well‐resourced referral infrastructures, conditions that are not consistently available across Indian oncology centres [8]. Narrative reviews, such as that by Bugshan et al. [19] provide valuable summaries of preventive and therapeutic strategies but do not deliver structured, validated, decision‐support tools suitable for routine oncology workflows.

At present, no validated manual in supportive dentistry specifically developed for the Indian oncology practice has been reported [20, 21]. In many Indian oncology centres, the limited integration of dental specialists into cancer care pathways necessitates practical tools that oncology clinicians can utilise before specialist referral [22]. The SDIM, therefore, addresses an implementation gap by translating guideline‐level recommendations into simplified algorithms, structured assessment parameters, and clearly defined referral pathways. This implementation‐focused orientation distinguishes the present manual from existing literature and positions it as a contextually adaptable tool for high‐volume, resource‐constrained oncology settings [23].

Although the manual showed strong content validity, several structural, workflow and system‐level challenges may affect the real‐world implementation of the SDIM in Indian oncology settings. High patient volumes, limited time for chairside assessment, and the absence of integrated dental services within oncology departments represent significant logistical barriers [22]. Fragmented referral pathways between oncology and dental services further impede the systematic delivery of oral supportive care [24]. Workforce‐related challenges, including a shortage of trained oncology dentists and limited awareness among general dentists regarding CROC, may reduce consistent adoption of the manual [23]. Successful implementation may be enhanced by incorporating the manual into existing oncology workflow and providing orientation sessions for members of the multidisciplinary team [15]. These implementation challenges should be directly addressed in future clinical piloting studies.

### 4.1. Strengths and Limitations

The major strength of this present study is the systematic, evidence‐based development process and the application of a content validity index (CVI)–based approach informed by Lawshe’s framework [25]. The combination of quantitative and qualitative expert refinement ensured statistical rigour with contextual relevance. The involvement of oral medicine specialists strengthened clinical applicability, and the structured, implementation‐oriented format enhances translational feasibility in resource‐constrained oncology settings.

A limitation of this study is that the expert panel consisted of 10 specialists, which, although methodologically acceptable for content validation studies, may limit generalisability. Additionally, all experts were specialists in oral medicine, rather than oncologists, nursing professionals, or other healthcare stakeholders, which may restrict interdisciplinary perspectives and limit the generalizability of the validation findings to other clinical specialities. Further studies should incorporate a multidisciplinary expert panel, including oncologists, oncology nurses, palliative care clinicians, and general dentists, to strengthen interdisciplinary validity. Moreover, the manual has undergone content validation but has not yet been clinically piloted or evaluated for outcome effectiveness. Lastly, the validation process was conducted within a limited geographic area, involving participants from two districts. Institutional clustering across the four academic institutions may have influenced consensus ratings, and further validation in broader, more geographically diverse settings may be necessary to improve generalisability.

## 5. Conclusion

This methodological content validation study demonstrated that the SDIM has strong content validity, clarity, and expert‐perceived feasibility for implementation in oncology settings. Feedback from the expert panel improved the manual’s organisation and usability while maintaining its evidence‐based content. The finalised manual provides a structured approach that may be suitable for routine oncology settings, pending clinical piloting and outcome evaluation. Further studies are recommended to evaluate its clinical effectiveness in practice.

**Nomenclature**
CT: Chemotherapy
CROC: Chemotherapy‐related oral complications
OM: Oral mucositis
MRONJ: Medication‐related osteonecrosis of the Jaw
SDIM: Supportive dentistry intervention manual
CVI: Content validity index
I‐CVI: Item‐level content validity index
S‐CVI: Scale level content validity index
CAMBRA: Caries management by risk assessment
QOL: Quality of life
WHO: World Health Organisation
MASCC/ISOO: Multinational association of supportive care in cancer and the international society of oral oncology
*k*: Modified kappa statistics.

## Author Contributions

**Nakshatra Shetty:** conceptualisation, writing – original draft, writing – review and editing. **Arun Ghoshal:** conceptualisation, methodology, writing – review and editing. **Adarsh Kudva:** methodology, supervision, writing – reviewing and editing. **Ananth Pai:** methodology, supervision, writing – review and editing. **Ravindranathan** **Vineetha:** supervision, writing – review and editing. **Sudeendra Prabhu:** writing – review and editing. **Anuja Damani:** writing – review and editing. **Krithika S**. **Rao:** writing – review and editing. **Abhinav Mishra:** writing – review and editing. **S. Gayatri:** writing – review and editing. **Kunal Das:** writing – review and editing. **Naveen Salins:** conceptualisation, methodology, supervision, writing – original draft, writing – reviewing and editing.

## Funding

This study did not receive any specific funding.

## Disclosure

All authors have read and approved the final version of the manuscript. Arun Ghoshal (corresponding author) had full access to all data in this study and takes full responsibility for the integrity and accuracy of the data and analysis.

## Ethics Statement

Ethical approval was not required because this study involved expert validation of a manual and did not include patient recruitment, clinical interventions, or the collection of identifiable personal or health‐related data.

## Consent

The authors have nothing to report.

## Conflicts of Interest

The authors declare no conflicts of interest.

## Supporting Information

Additional supporting information can be found online in the Supporting Information section.

## Supporting information


**Supporting Information** Table S1: Representative section of the supportive dentistry intervention manual (SDIM). This table presents the structure and major content domains of the SDIM, including basic oral care protocols, management of chemotherapy‐related oral complications, practical tools, referral pathways and bibliography. Table S2: Sample management table from the SDIM: oral hygiene instructions. This table provides a representative example of the evidence‐based oral hygiene recommendations included in SDIM. Figure S1: Flowchart illustrating the basic oral care protocol included in the SDIM. This figure presents the stepwise oral care pathway recommended for patients receiving chemotherapy, including oral assessment, routine oral hygiene practices, patient and caregiver education, monitoring for warning signs and referral when indicated.

## Data Availability

The authors confirm that the data supporting the findings of this study are available within the article and/or its Supporting Information. A summary of the validated manual is provided as Supporting Information. The complete manual is not publicly available as it constitutes the primary intellectual content developed by the authors, but may be shared by the corresponding author upon reasonable request for academic and research purposes.
