# Supplementary material for: Structured Supportive Dentistry Intervention Manual for the Oral Complications in Patients With Cancer Receiving Chemotherapy: Development and Content Validation
Source: Int J Dent. 2026 Aug 7;2026:1554891. doi: 10.1155/ijod/1554891 (PMC13449172; doi:10.1155/ijod/1554891)
Supplement: Supplementary file 1 [file IJOD-2026-1554891-s001.docx]

**Supplementary files– Representative section of the SDIM**

**Supplementary Table S1: Representative section of the supportive dentistry intervention manual (SDIM).**

1. **Introduction and Scope**

2. **Basic Oral Care Protocol**
   2.1. Oral hygiene instructions
   2.2. Patient and caregiver education and follow-up
   2.3. Warning signs

3. **Soft tissue-related oral complications**
   3.1. Xerostomia
   3.2. Oral Mucositis
   3.3. Taste Disturbance (Dysgeusia)
   3.4. Burning Mouth
   3.5. Oral Pigmentation

4. **Oral Infections**
   4.1. Fungal infection
   4.2. Candidiasis
   4.3. Viral infections
a. Herpes Simplex Infection
b. Varicella Zoster Infection

5. **Hard tissue-related oral complications**
   5.1. Dental Caries
   5.2. Pulpoperiapical Lesions
   5.3. Medication Related Osteonecrosis of Jaw (MRONJ

6. **Practical tools**
   6.1. Oral cavity examination
   6.2. Referral pathway

7. **Bibliography**

**Supplementary Table S2: Sample management table from SDIM: Oral Hygiene instructions.**

| Element | Recommended approach | Rationale |
| --- | --- | --- |
| Tooth brushing (3,9) | - Brush teeth and gums 2 or 3 times a day for 2-3 minutes using soft bristled toothbrush, with vertical motion for anterior teeth and circular motion for posterior teeth. - Air-dry the toothbrush between each use. - Replace the toothbrush once every 3 months. - Foam swab or gauze may be used when a soft toothbrush is not tolerated, or when the patient has neutropenia (<500/mm3) or thrombocytopenia (<40, 000/mm3) | Reduces plaque, infection risk, and oral mucositis severity while minimising trauma to the oral mucosa. |
| Flossing/ interdental brushing (9) | - Gentle flossing once daily using waxed dental floss by sliding it between each tooth and along both sides or use water flossing for tight contacts. - Interdental brushes (interdental gap ≥ 1mm) | Prevent interdental plaque and gingivitis and avoid mucosal trauma and bleeding. |
| Tongue cleaning | - Gentle cleaning the dorsal tongue once daily using soft toothbrush or a tongue cleaner/scraper, moving from back to front, immediately after tooth brushing. - Avoid vigorous scraping if mucositis or bleeding tendency is present. | Reduce tongue coating and debris accumulation, improves taste and bad breath, and aids differentiation from candidiasis, which typically present as a wipeable plaque with an erythematous base. |
| Toothpaste (6) | - Non-mint, detergent-free toothpaste for ulcers/xerostomia - Paediatric toothpaste if adult options unavailable - Fluoride ≥1100 ppm | Prevent caries and plaque without irritating the mucosa. |
| Mouth rinses (9,10) | - Saline mouthwash: 4-6 times a day (½–1 teaspoon of common salt mixed in 1 glass (200–250 ml) of boiled and cooled drinking water). A fresh supply should be made daily. - Alcohol free Chlorhexidine 0.12% mouthwash to treat gingivitis. | Provide comfort, cleanliness, and mucositis control. |
| Denture care (11) | - Brush and rinse using a soft-bristle toothbrush/denture cleaning brush. Rinse after every meal. - Soak in normal water or preferably 0.12% chlorhexidine mouthwash overnight (avoid hot water). | Prevent fungal infection. |
| Hydration/moisturization (11) | - Frequent sipping of water, - Lip lubrication. | Manage dry mouth, improve comfort and function, and lower risk for dental caries. |

**Supplementary figure S1: Flowchart illustrating the Basic oral care protocol included in the SDIM.**
